# Supplementary figures and images for: A tertiary center experience of multiple myeloma patients with COVID-19: lessons learned and the path forward
Source: J Hematol Oncol. 2020 Jul 14;13:94. doi: 10.1186/s13045-020-00934-x (PMC7359431; doi:10.1186/s13045-020-00934-x)

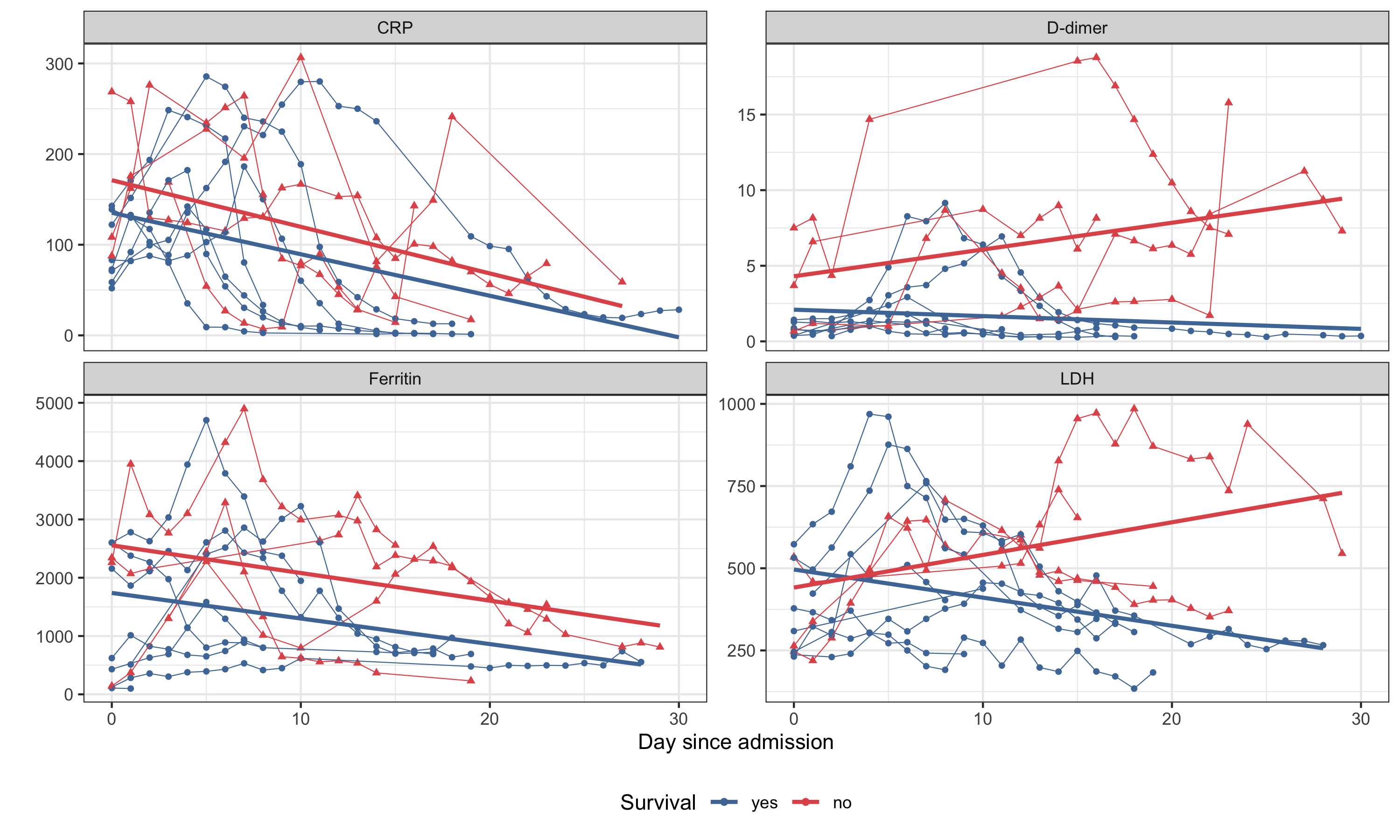

Supplement: Supplementary file 1 — Additional file 1: Figure S1. Evolution of selected inflammatory biomarkers in a subset of patients (n = 12) hospitalized at the Mount Sinai Hospital for which the data was available. Different measurements from the same patient are connected. A linear regression line is plotted for the subgroup of patients that survived (blue, n = 8) and died (red, n = 4), respectively. [file 13045_2020_934_MOESM1_ESM.png]
